# Surgical approaches in total hip arthroplasty do not influence the bacterial spectrum of acute postoperative periprosthetic joint infections

**DOI:** 10.5194/jbji-10-385-2025

**Published:** 2025-10-24

**Authors:** Jonas Tumler, Dominic Simon, Gautier Beckers, Alexander C. Paulus, Boris M. Holzapfel, Jörg Arnholdt

**Affiliations:** 1 Department of Orthopaedics and Trauma Surgery, Musculoskeletal University Center Munich (MUM), University Hospital, LMU Munich, Marchioninistr. 15, 81377 Munich, Germany; 2 Orthopaedic Specialist Center Weilheim, Johann-Baur-Str. 5, 82362 Weilheim, Germany

## Abstract

**Introduction**: With the increasing number of primary total hip arthroplasties (THAs), the incidence of associated complications has risen, with periprosthetic joint infection (PJI) being among the most severe. The influence of surgical approach on infection risk remains debated; however, its effect on the microbiological profile of PJIs is not well examined. This study aimed to evaluate whether the primary surgical approach affects the spectrum of microorganisms involved in acute postoperative periprosthetic hip joint infections in a retrospective single-center cohort. **Methods**: A total of 76 patients who underwent revision surgery for PJI following THA between January 2013 and June 2024 were retrospectively reviewed. After applying exclusion criteria, patients were categorized based on the initial surgical approach: lateral vs. direct anterior/anterolateral. The microbiological spectrum was compared between groups using Fisher's exact test. Demographic characteristics and their associations with surgical approach and pathogen type were also analyzed. **Results**: No significant differences were found in the microbiological spectrum between surgical approaches. Similarly, there was no significant correlation between surgical approach and the occurrence of Gram-positive or Gram-negative organisms. Body mass index (BMI) was not associated with variations in pathogen distribution, suggesting that obesity does not influence the microbiological profile of PJI. **Discussion**: These findings suggest that the microbiological characteristics of acute postoperative PJI are independent of the primary surgical approach. Minimally invasive anterior techniques do not appear to carry an increased microbiological risk. Additionally, BMI does not influence the microbial spectrum of infection. **Level of evidence**: this is a retrospectively registered cohort study with a Level III level of evidence.

## 1 Introduction

Periprosthetic joint infection (PJI) remains a serious complication following primary total hip arthroplasty (THA). While infection rates in primary THA have decreased over the past 2 decades (Zeng et al., 2023; Phillips et al., 2006) to approximately 1 %–2 % nowadays (Chalmers et al., 2020; Ebrahimzadeh et al., 2023), the incidence remains about 5 % in revision procedures (Zeng et al., 2023; Holinka and Windhager, 2016). PJI is associated with significant morbidity and mortality (Shahi et al., 2017; Boddapati et al., 2018). Additionally, its complex and prolonged treatment (Li et al., 2021) poses a substantial economic burden on healthcare systems worldwide (Premkumar et al., 2021; Kamath et al., 2015; Zardi and Franceschi, 2020; Patel and Golwala, 2023).

With rising life expectancy and shifting population demographics (Kamath et al., 2015), the demand for elective THA and hip surgeries for geriatric-trauma-related fractures is projected to increase. Although advancements in perioperative protocols have reduced individual surgical risks, the overall increase in procedural volume has led to a higher absolute number of complications. This highlights the need for ongoing efforts to minimize complication rates, reduce the burden on healthcare systems, and enhance patient outcomes (Abuelnour et al., 2025). Due to its limitations, including hip abductor weakening and increased blood loss, the lateral approach has been largely abandoned (Patel and Golwala, 2023; Docter et al., 2020). In contrast, the direct anterior approach (DAA) has gained widespread popularity in recent years, offering advantages including reduced muscular and neural impairment (Moerenhout et al., 2020; Driesman and Yang, 2023; Chen et al., 2020), faster recovery, minimized soft tissue trauma, and reduced postoperative pain (Driesman and Yang, 2023).

Previous works have examined the relationship between PJI and various surgical approaches (O'Connor et al., 2021; Ilchmann et al., 2016). However, little is known about the impact of surgical approach on the microbial spectrum in PJIs, with current analyses demonstrating contradictory results (Aichmair et al., 2022). Dockery et al. (2023) reported higher infection rates in patients undergoing THA using a DAA compared to non-anterior approaches, while O'Connor et al. (2021) found no significant difference in infection rates based on surgical approach (O'Connor et al., 2021; Dockery et al., 2023).

Given the proximity of the anterior approach to the groin, a highly colonized and humid area (Grice and Segre, 2011), the question arises as to whether the microbial spectrum of periprosthetic infections in primary THA varies with the surgical approach. Answering this question could influence perioperative antibiotic prophylaxis and infection management, potentially reducing the incidence of PJI and improving its treatment.

**Figure 1:**
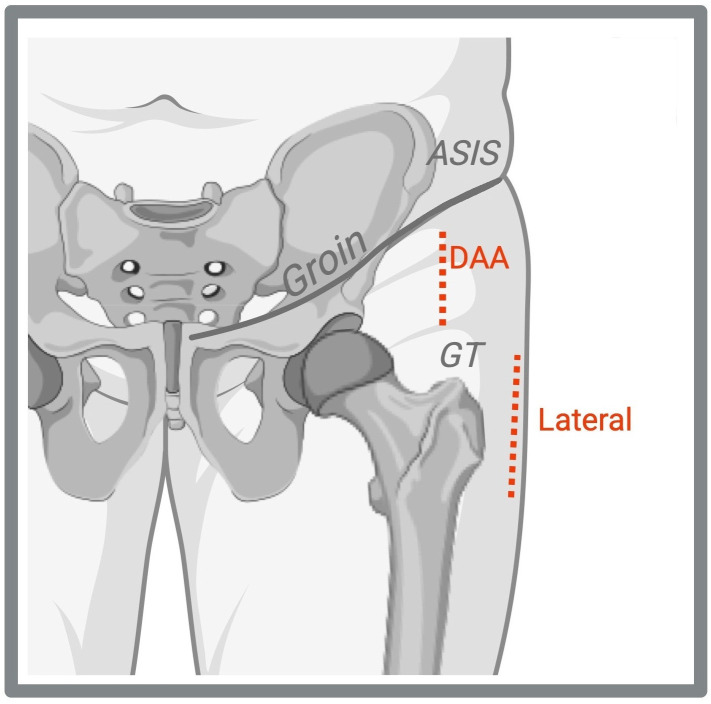
Skin incisions for total hip arthroplasty. The direct anterior approach (DAA) is localized in the groin, just lateral and distal to the anterior superior iliac spine (ASIS). The lateral approach is positioned on the proximal lateral thigh over the greater trochanter (GT). Created with https://www.biorender.com/ (last access: 22 August 2025).

The DAA uses an intermuscular and internervous plane between the tensor fasciae latae and sartorius, with the skin incision placed in the groin region just lateral and distal to the anterior superior iliac spine (ASIS). This muscle-sparing route may enable faster early recovery, though it has a steep learning curve and carries a risk of injury to the lateral femoral cutaneous nerve. In contrast, the lateral approach involves incisions on the proximal lateral thigh over the greater trochanter, providing excellent exposure, however requiring splitting or detaching parts of the gluteus medius and/or minimus, which can lead to postoperative abductor weakness (Fig. 1).

This study aims to assess the microbial spectrum following THA in relation to the surgical approach and patient-specific data.

1. We hypothesize that patients undergoing THA via DAA/anterolateral approaches may exhibit a different microbiological spectrum in PJI compared to those undergoing lateral approaches.
2. Based on the research by Böni et al. (2018) and Maurer et al. (2021), we anticipate higher infection rates associated with *Cutibacterium avidum* and Gram-negative pathogens in DAA patients (Corvec, 2018).
3. Furthermore, we hypothesize that obese patients exhibit a different microbiological spectrum in PJI compared to non-obese patients.
4. Additionally, we expect a broader presence of anaerobic bacteria, particularly among obese patients (Watts et al., 2015; Ren et al., 2021), due to their colonization in the overlapping groin fold (Maurer et al., 2021; Böni et al., 2018; Karlsson et al., 2024).

## 2 Methods

We conducted a single-center retrospective cohort study at a university hospital to compare the microbiological spectrum of PJI between patients who underwent primary THA via the direct anterior/anterolateral approach and those operated on using the lateral approach. It is important to note that this is a single-center study, which limits the generalizability of its findings, and that the small number of cases carries the risk overlooking differences in the germ spectrum across surgical approaches. Furthermore, our cohort included both in-house and referred patients.

**Figure 2:**
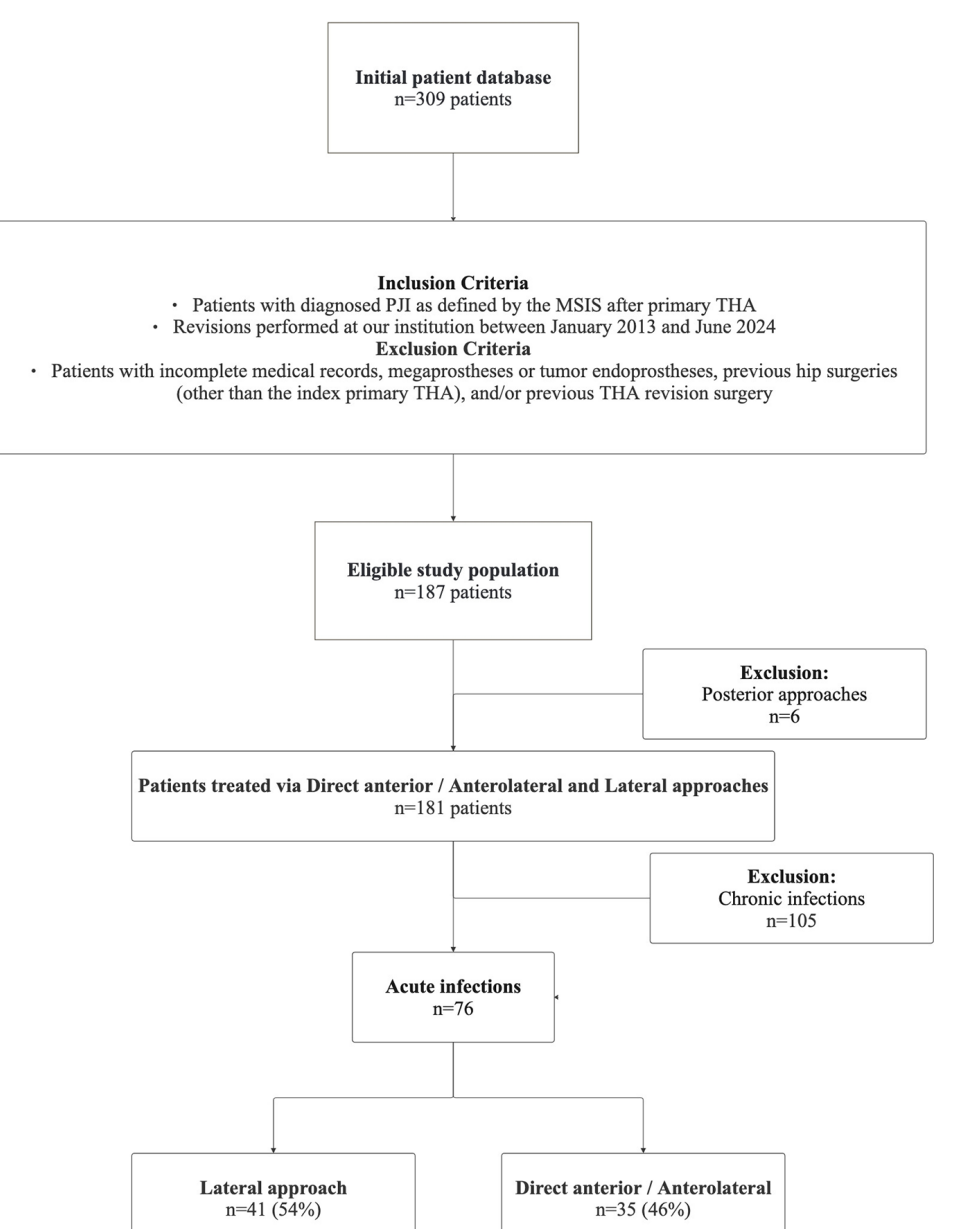
Flowchart diagram of patient inclusion and exclusion criteria. Abbreviations: PJI – periprosthetic joint infection, MSIS – musculoskeletal infection society, THA – total hip arthroplasty, DAA – direct anterior approach.

A total of 309 patients were initially identified using the International Statistical Classification of Diseases and Related Health Problems (ICD) code T84.5, which denotes infection and inflammatory reaction due to internal joint prosthesis. After applying the exclusion criteria, 187 eligible patients who were treated for PJI between January 2013 and June 2024 were identified. After excluding six posterior approaches and 105 chronic infections, the final study group comprised 76 patients. A flowchart summarizing the inclusion and exclusion criteria is presented in Fig. 2.

Additionally, patients with negative microbiological findings but presenting clinical symptoms and typical laboratory parameters, such as elevated cell counts from diagnostic punctures, were classified as microbiologically negative infections. PJIs were retrospectively confirmed according to the Musculoskeletal Infection Society (MSIS) criteria (Parvizi et al., 2018). Up to five microbiological samples were incubated for 14 d at the Institute of Microbiology prior to analysis. This protocol remained unchanged throughout the investigation period. The project with project number 23-0905 was approved by the local Ethics Committee. Data were obtained from patients' electronic medical records.

**Table 1:** Demographic and clinical parameters.

	Overall	Lateral	DAA/anterolateral	p
	N=76	N=41	N=35	value
Gender (%)				
men	38 (50.0)	20 (48.8)	18 (51.4)	n.s.
women	38 (50.0)	21 (51.2)	17 (48.6)	n.s.
Mean age (years)	73.7±11.2 (46–94)	75.5±11.3	71.5±10.8 (46–91)	n.s.
Mean BMI	27.2±6.5 (15–52)	27.2±6.7	27.3±6.4 (18–52)	n.s.
ASA score	2.8±0.5 (2–4)	2.9±0.5 (2–4)	2.8±0.5 (2–4)	n.s.
CCI	2.5±2.0 (0–8)	2.6±2.0 (0–7)	2.3±2.1 (0–8)	n.s.
Monobacterial infection (%)	48/73 (65.8)	28/40 (70.0)	20/33 (61.0)	n.s.
Anticoagulants (%)	33/73 (45.2)	20/39 (51.3)	13/33 (39.0)	n.s.
Diabetes (%)	17/71 (23.9)	11/40 (27.5)	6/31 (19.4)	n.s.
Immunosuppression (%)	7/76 (9.2)	2/41 (4.9)	5/35 (14.3)	n.s.
Smoking/alcohol (%)	27/73 (37.0)	14/40 (35.0)	13/33 (39.4)	n.s.
Time after implantation (days)	23.7±11.4 (6–42)	23.0±11.8 (6–42)	24.7±11.0 (6–42)	n.s.
Type of implantation (%)				
cemented	23/76 (30.3)	17/41 (41.5)	6/35 (17.1)	
uncemented	42/76 (55.3)	17/41 (41.5)	25/35 (71.4)	
hybrid fixation	11/76 (14.5)	7/41 (17.1)	4/35 (11.4)	

Abbreviations: BMI – body mass index, ASA – American Society of Anesthesiologists, CCI – Charlson Comorbidity Index. For gender, monobacterial infection, anticoagulants, diabetes, immunosuppression, smoking/alcohol and type of implantation, the numbers represent N (%). For mean age, mean BMI, ASA, CCI and time after implantation, the numbers represent mean ± standard deviation (range).

Infections occurring within 6 weeks of the primary THA were classified as acute, whereas those developing thereafter were considered chronic (Xu et al., 2019). Acute infections are more likely to be influenced by the surgical approach and are therefore more relevant to our research question than chronic infections (Izakovicova et al., 2019).

Monobacterial infections were defined as those caused by a single pathogen, while polybacterial infections involved multiple pathogens.

Patient characteristics recorded included age, gender, type of infection (acute/chronic), BMI, surgical approach, Charlson Comorbidity Index (CCI), American Society of Anesthesiologists (ASA) classification, diabetes mellitus (DM), time between implantation and PJI, treatment (DAIR/Spacer/Girdlestone), risk factors such as smoking and/or alcohol consumption, anticoagulation, and immunosuppression. Additionally, data on the type of implantation (cemented/uncemented/partially cemented) and the number and type of microbiological findings were collected (Table 1).

- *Primary endpoint*: To assess the microbiological spectrum of periprosthetic hip joint infections in relation to the primary surgical approach.
- *Secondary endpoints*: To assess the relationship between obesity and the microbiological spectrum, the entire cohort was divided into two groups: obese (BMI ≥ 30) and non-obese (BMI < 30) patients (Panuganti et al., 2024).
- *Statistical analysis*: Categorical variables are presented as frequencies and percentages, while continuous variables are expressed as mean ± standard deviation (SD). Pearson's chi-squared test was used to compare proportions, or Fisher's exact test was employed if any expected cell count was less than 5.

Because of multiple testing, the Bonferroni–Holm adjustment of the p value was performed. A p value below 0.05 was considered statistically significant. Statistical analyses were performed with SPSS (Version: 29.0.2.0, SPSS Inc., Chicago, IL, USA). Data preparation and initial calculations were performed using Microsoft Excel version 16.102 (Microsoft 365) for macOS (Microsoft Corp., 2023; IBM Corp., 2023). Document preparation was done using Microsoft Word version 16.102 (Microsoft 365) for macOS (Microsoft Corp., 2023).

## 3 Results

In the cohort of 76 patients with acute infections, the gender distribution was balanced, with 38 women and 38 men. The average BMI in the cohort was 27.2±6.5 , and the mean ASA score was 2.8±0.5 . Surgical approaches included the lateral approach in 54.0 % of cases ( 41/76 ), the DAA in 28.9 % ( 22/76 ), and the anterolateral approach in 17.1 % ( 13/76 ).

**Figure 3:**
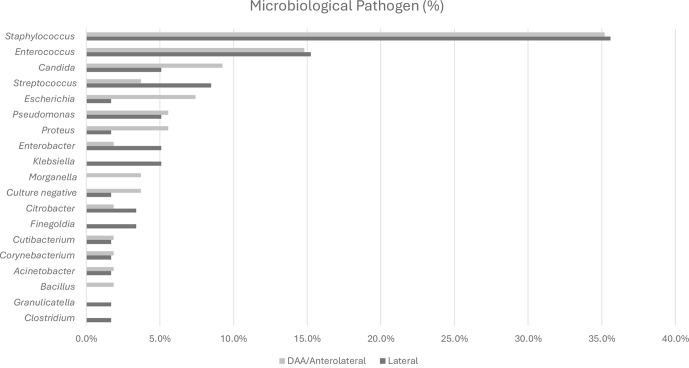
Microbiological spectrum in periprosthetic hip joint infections ( n=76 ). Abbreviations: DAA – direct anterior approach.

Among the infections, 65.8 % (48/73) were monobacterial, while 34.2 % (25/73) were polymicrobial. The average time from the index hip surgery to the onset of PJI was 23.7±11.4 d (range: 6–42). The CCI averaged 2.5±2.0 across the cohort, with no significant differences observed between surgical approaches: 2.6±2.0 for the lateral approach, 2.7±2.3 for the DAA, and 1.7±1.5 for the anterolateral approach. Despite the slightly lower CCI in the anterolateral group, overall comorbidity profiles were comparable across all groups. Demographic details are summarized in Table 1.

### 3.1 Primary endpoint

The most commonly detected pathogen across the entire acute infection cohort was *Staphylococcus epidermidis*, identified in 17 cases (15 %). These findings are summarized in Fig. 3. The second-most-frequent pathogen was *Staphylococcus aureus*, found in 16 cases (14.2 %). *Staphylococcus epidermidis* was the most prevalent pathogen in the lateral approach cohort, representing 15.3 %, while *Staphylococcus aureus* was most prevalent in the DAA/anterolateral cohort, accounting for 16.7 %. These findings are summarized in Table 2.

1. No significant association was observed between the bacterial spectrum and the surgical approach. Fisher's exact test yielded p values > 0.05 for all pathogen types and surgical approaches, both before and after adjustment.
2. *Cutibacterium acnes* and *Cutibacterium avidum* were each detected only once, with *C. acnes* identified in the lateral group and *C. avidum* in the DAA/anterolateral group.
  Furthermore, no significant correlation was found between the surgical approach and the occurrence of Gram-positive or Gram-negative pathogens ( p=0.199 for Gram-positive, p=0.248 for Gram-negative). Gram-positive pathogens were more frequently identified in both the lateral and DAA/anterolateral cohorts compared to Gram-negatives. However, their distribution showed no significant variation across the different surgical approaches. Similarly, Gram-negative pathogens were evenly distributed among the approaches despite being less commonly detected.
3. Additionally, no increased prevalence of anaerobic bacteria was observed among obese patients in our cohort.

### 3.2 Secondary endpoint

We found no significant association between obesity and the microbiological spectrum in PJIs following THA. Initially, before applying the Bonferroni–Holm adjustment, *Corynebacterium* and *Proteus* showed a significant association, with *Corynebacterium* being more prevalent in non-obese patients and *Proteus* detected exclusively in obese patients with a BMI over 30. However, after adjusting for multiple testing, all results became insignificant, indicating weak influence of BMI on the microbiological spectrum. Additionally, *Cutibacterium* did not show any significant relationship with BMI. These findings are summarized in Table 3.

**Table 2:** Summary table: Fisher's exact test on dependence between surgical approach and microbes (in groups).

	Microbial	Overall	Lateral	DAA/anterolateral	p value	p value
	pathogen	N (%)				(adjusted)
	*Staphylococcus*	40 (35.4)	21	19	0.525	n.s.
	*Enterococcus*	17 (15.0)	9	8	1	n.s.
Gram-positive	*Streptococcus*	7 (6.2)	5	2	0.442	n.s.
	*Corynebacterium*	2 (1.8)	1	1	1	n.s.
	*Bacillus*	1 (0.9)	0	1	0.461	n.s.
	*Granulicatella*	1 (0.9)	1	0	1	n.s.
	*Pseudomonas*	6 (5.3)	3	3	1	n.s.
	*Escherichia*	5 (4.4)	1	4	0.174	n.s.
	*Enterobacter*	4 (3.5)	3	1	0.62	n.s.
Gram-negative	*Proteus*	4 (3.5)	1	3	0.329	n.s.
	*Citrobacter*	3 (2.7)	2	1	1	n.s.
	*Klebsiella*	3 (2.7)	3	0	0.245	n.s.
	*Acinetobacter*	2 (1.8)	1	1	1	n.s.
	*Morganella*	2 (1.8)	0	2	0.209	n.s.
	*Cutibacterium*	2 (1.8)	1	1	1	n.s.
Anaerobes	*Finegoldia*	2 (1.8)	2	0	0.496	n.s.
	*Clostridium*	1 (0.9)	1	0	1	n.s.
Fungi	*Candida*	8 (7.1)	3	5	0.827	n.s.
Culture-negative	No detected pathogen	3 (2.7)	1	2	0.592	n.s.

Abbreviations: DAA – direct anterior approach, n.s. – non significant ( p>0.05 ).

**Table 3:** Summary table: Fisher's exact test on dependence between obesity and microbes.

	Microbial pathogen	Overall	No BMI	Obesity no	Obesity yes	p value	p value
				BMI < 30	BMI ≥ 30		adjusted
	*Staphylococcus*	40	0	31	9	0.962	>0.999
	*Enterococcus*	17	1	12	4	0.313	>0.999
Gram-positive	*Streptococcus*	7	1	5	1	0.101	>0.999
	*Corynebacterium*	2	1	1	0	0.026	0.338
	*Bacillus*	1	0	1	0	1	>0.999
	*Granulicatella*	1	0	1	0	1	>0.999
	*Pseudomonas*	6	0	2	4	0.077	>0.999
	*Escherichia*	5	0	5	0	0.366	>0.999
	*Enterobacter*	4	0	3	1	1	>0.999
Gram-negative	*Proteus*	4	0	0	4	0.004	0.056
	*Citrobacter*	3	0	2	1	1	>0.999
	*Klebsiella*	3	0	3	0	0.584	>0.999
	*Acinetobacter*	2	0	1	1	0.46	>0.999
	*Morganella*	2	0	1	1	0.46	>0.999
	*Cutibacterium*	2	0	1	1	0.46	>0.999
Anaerobes	*Finegoldia*	2	0	2	0	1	>0.999
	*Clostridium*	1	0	0	1	0.263	>0.999
Fungi	*Candida*	8	0	4	4	0.098	>0.999
Culture-negative	No detected pathogen	3	0	1	2	0.189	>0.999

Abbreviations: BMI – body mass index.

## 4 Discussion

### 4.1 Primary endpoint

The main finding of this study is that there is no significant correlation between the surgical approach and the spectrum of pathogens identified in PJI following THA. This suggests that the choice of surgical approach does not substantially influence the microbiological profile of acute PJIs or that other factors may play a more dominant role. Likewise, no increased frequency of *Cutibacterium* species was observed in the DAA/anterolateral cohort compared to the lateral group.

Therefore, our findings contrast with those of previous studies, which were limited by small sample sizes and lacked robust statistical significance (Aichmair et al., 2022; Ilchmann et al., 2016; Buchalter et al., 2020). Notably, two similar studies are of interest: one by Buchalter et al. (2020), which found that the pathogen profile in THA PJIs differs by approach, with more Gram-negative infections in DAA compared to non-DAA approaches (Buchalter et al., 2020), and another by Aichmair et al. (2022), which reported higher rates of *Cutibacterium avidum* in the DAA group (22.2 % vs. 2.8 %, p=0.028 ) compared to lateral approaches (Aichmair et al., 2022).

This study's findings suggest that, compared to other approaches, the DAA does not confer a disadvantage in terms of the pathogen spectrum. Furthermore, data from the Australian Orthopaedic Association National Joint Replacement Registry (AOANJRR) suggest a low cumulative incidence of septic revision procedures for primary hip replacement using the anterior approach (Lewis et al., 2024). Nonetheless, concerns had been raised regarding the proximity of the DAA to the groin, an area densely populated with bacteria (Holinka and Windhager, 2016; Böni et al., 2018; Grice and Segre, 2011), suggesting this approach might increase infection risks or alter the pathogen profile. This concern stems from the moist environment in the groin fold, particularly in overweight patients, which may facilitate the growth of specific pathogens such as *Cutibacterium*, potentially leading to a higher risk of PJI compared to lateral or posterior approaches (Böni et al., 2018; Karlsson et al., 2024; Grice and Segre, 2011).

While several hypotheses propose that the pathogen spectrum may vary depending on the surgical approach, there are also plausible reasons why it may remain consistent. For instance, the skin incision used in the DAA can be positioned relatively laterally while still permitting access to the hip joint through the Hueter interval. This overlap in surgical access may reduce anatomical differences between the DAA and lateral approaches, potentially resulting in a similar pathogen profile.

Additionally, there are other reasons why the microbial spectrum might not differ significantly between approaches. First, human skin is naturally colonized by a diverse microbiome, which is present across all skin regions regardless of the incision site. (Watts et al., 2015; Grice and Segre, 2011). Common PJI-causing pathogens, such as *Staphylococcus aureus* and coagulase-negative *Staphylococci*, are part of this normal skin microbiome (Grice and Segre, 2011; Tande and Patel, 2014). Second, the primary sources of microbial contamination during surgery, such as operating room personnel, air quality, and surgical instruments, are generally consistent across surgical approaches, further supporting our findings. Third, individual risk factors such as diabetes, obesity, or immunosuppression, which elevate the likelihood of infection, are independent of the surgical approach (Watts et al., 2015; Alamanda and Springer, 2018). Furthermore, the use of standardized aseptic techniques and infection prevention protocols, such as skin disinfection, sterile instruments, and prophylactic antibiotics, is consistent across approaches, potentially contributing to similar microbial profiles in PJIs.

It is well established that microbial colonization in humans varies by anatomical region, influenced by factors such as skin folds and proximity to the genitourinary and gastrointestinal tracts (Maurer et al., 2021; Grice and Segre, 2011; Haverkamp et al., 2011). These areas, characterized by higher temperatures and humidity, provide an ideal environment for microorganisms that thrive in moist conditions (Chalmers et al., 2020). Metagenomic analyses have shown that *Staphylococcus* and *Corynebacterium* species are the most prevalent in these regions (Maurer et al., 2021; Böni et al., 2018; Grice and Segre, 2011). However, several factors, including the use of iodized antiseptic drapes and draping techniques, surgical positioning, preoperative and intraoperative antibiotic prophylaxis, preoperative washing, and the minimal distance between anterior and lateral approaches, may explain why the pathogen spectrum does not differ significantly between surgical techniques.

### 4.2 Secondary endpoint

This study did not find a significant correlation between BMI and the detected microbial pattern. While obesity is a recognized risk factor for PJI after THA (Watts et al., 2015; Haverkamp et al., 2011), it remains unclear as to whether it also influences the pathogen spectrum. This study did not observe a significant correlation. However, the p values suggest a tendency toward *Proteus*. Moreover, all patients in whom *Proteus* was detected were obese (BMI ≥ 30). However, obese individuals typically present with a greater burden of comorbidities compared to non-obese patients, which could theoretically contribute to higher complication rates (Yoon et al., 2024) and potentially explain observed similarities in pathogen profiles (Watts et al., 2015; Haverkamp et al., 2011; Vasarhelyi and MacDonald, 2012).

Nonetheless, the limited sample size must be acknowledged. Larger studies are necessary to investigate this question in greater detail.

### 4.3 Limitations

This study provides valuable insights into the role of the surgical approach in PJI and highlights key risk factors in this complex area. Despite its contributions, several limitations must be considered to interpret the findings and guide future research. Furthermore, validation in larger, multicenter cohorts will be necessary before any modifications to prophylactic or empiric therapy guidelines can be recommended.

A primary limitation is the relatively small sample size, a consequence of the low prevalence (1 %–2 %) of PJIs. With only 76 cases included, the limited statistical power increases the risk of type II errors, thereby restricting the generalizability of the results to larger populations.

Additionally, due to the limited sample size in some subgroups, we relied on unadjusted analyses, which do not account for potential confounding factors. While multivariable modeling would be methodologically preferable to isolate the effect of the surgical approach from variables such as BMI or comorbidities, the small number of events precluded such analyses. As a result, the observed associations should be interpreted with caution.

Another consideration is the heterogeneity within the dataset. Patients were treated by multiple surgeons with varying techniques and levels of experience, which may introduce variability and complicate attribution of outcomes to specific surgical practices. However, this diversity also enhances the external validity of the results and reflects the range of real-world clinical practice, thereby improving generalizability to the broader orthopedic community. Similarly, while differences in microbiological detection methods across laboratories may affect the consistency of pathogen identification, they also mirror routine clinical settings and make the findings more applicable to everyday practice.

Moreover, as a university hospital and tertiary referral center, the institution treated patients from a wide network of peripheral hospitals. This wide catchment increases the diversity of patient and treatment characteristics, which may introduce variability but also contributes to the study's relevance across varied clinical contexts.

However, relatively few culture-negative cases were present in our cohort (3 of 76 patients; 2 in DAA/anterolateral and 1 in lateral), which may limit complete characterization of the microbiological spectrum.

Lastly, this study only analyzed differences in pathogens for acute infections. Therefore, the absence of differences in the bacterial spectrum observed cannot be generalized to chronic infections. Since *Cutibacteria* are frequently associated with chronic infections (Warne et al., 2024), this study, which focuses solely on acute infections, only permits a limited assessment of the distribution of *Cutibacterium* species in relation to the surgical approach.

## 5 Conclusion

This study demonstrates a comparable microbiological spectrum in acute PJI following both direct anterior/anterolateral and lateral approaches during primary total hip arthroplasty. Modern, minimally invasive anterior approaches did not show a significantly higher incidence of infections caused by anaerobic or Gram-negative bacteria, supporting their microbiological safety despite proximity to the groin area. Furthermore, no significant association was found between obesity and the presence of specific pathogens.

## Data Availability

Data used in this work are available from the corresponding author upon reasonable request.

## Appendix A

**Table A1:** Summary table: Fisher's exact test on dependence between approach and microbes.

	Microbial pathogen	Overall	Lateral	DAA/	p value	p value
		N (%)		anterolateral		(adjusted)
	*Staphylococcus epidermidis*	17 (15.0)	9	8	1	n.s.
	*Staphylococcus aureus*	16 (14.2)	7	9	0.407	n.s.
	*Enterococcus faecalis*	9 (8.0)	6	3	0.494	n.s.
	*Enterococcus faecium*	8 (7.1)	3	5	0.459	n.s.
	*Staphylococcus capitis*	4 (3.5)	3	1	0.620	n.s.
	*Staphylococcus haemolyticus*	3 (2.7)	2	1	1	n.s.
	*Streptococcus dysgalactiae*	2 (1.8)	1	1	1	n.s.
Gram-positive	*Streptococcus oralis*	2 (1.8)	1	1	1	n.s.
	*Bacillus* sp.	1 (0.9)	0	1	0.461	n.s.
	*Corynebacterium striatum*	1 (0.9)	1	0	0.461	n.s.
	*Corynebacterium tubercolostaticum*	1 (0.9)	0	1	1	n.s.
	*Granulicatella adiacens*	1 (0.9)	1	0	1	n.s.
	*Streptococcus agalactiae*	1 (0.9)	1	0	1	n.s.
	*Streptococcus bovis*	1 (0.9)	1	0	1	n.s.
	*Streptococcus pneumoniae*	1 (0.9)	1	0	1	n.s.
	*Pseudomonas aeruginosa*	6 (5.3)	3	3	1	n.s.
	*Escherichia coli*	5 (4.4)	1	4	0.174	n.s.
	*Enterobacter cloacae*	4 (3.5)	3	1	0.62	n.s.
	*Proteus mirabilis*	4 (3.5)	1	3	0.329	n.s.
	*Citrobacter koseri*	2 (1.8)	2	0	0.496	n.s.
Gram-negative	*Klebsiella pneumoniae*	2 (1.8)	2	0	0.496	n.s.
	*Morganella morganii*	2 (1.8)	0	2	0.209	n.s.
	*Acinetobacter baumannii*	1 (0.9)	0	1	0.461	n.s.
	*Acinetobacter dijkshoorniae*	1 (0.9)	1	0	1	n.s.
	*Citrobacter freundii*	1 (0.9)	0	1	0.461	n.s.
	*Klebsiella oxytoca*	1 (0.9)	1	0	1	n.s.
	*Finegoldia magna*	2 (1.8)	2	0	0.496	n.s.
Anaerobes	*Clostridium disporicum*	1 (0.9)	1	0	1	n.s.
	*Cutibacterium acnes*	1 (0.9)	1	0	1	n.s.
	*Cutibacterium avidum*	1 (0.9)	0	1	0.461	n.s.
	*Candida albicans*	3 (2.7)	2	1	1	n.s.
Fungi	*Candida glabrata*	3 (2.7)	1	2	0.592	n.s.
	*Candida parapsilosis*	1 (0.9)	0	1	0.461	n.s.
	*Candida species*	1 (0.9)	0	1	0.461	n.s.
Culture-negative		3 (2.7)	1	2	0.592	n.s.

Abbreviations: DAA – direct anterior approach, n.s. – non-significant ( p>0.05 ).

## Appendix B

**Table B1:** Distribution of detected germs by approach with 95 % confidence intervals.

	Microbial pathogen	Overall	95 %-CI	Lateral	95 %-CI	DAA/	95 %-CI
		N (%)		N (%)		anterolateral	
						N (%)	
	*Staphylococcus*	40 (35.4)	26.6–45.0	21 (52.5)	36.1–68.5	19 (47.5 %)	36.6–71.2
	*Enterococcus*	17 (15.0)	9.0–23.0	9 (52.9)	27.8–77.0	8 (47.1 %)	10.4–40.1
Gram-positive	*Streptococcus*	7 (6.2)	2.5–12.3	5 (71.4)	29.0–96.3	2 (28.6 %)	0.7–19.2
	*Corynebacterium*	2 (1.8)	0.2–6.2	1 (50.0)	1.3–98.7	1 (50.0 %)	0.1–14.9
	*Bacillus*	1 (0.9)	0.0–4.8	0 (0.0)	0.0–97.5	1 (100.0 %)	0.1–14.9
	*Granulicatella*	1 (0.9)	0.0–4.8	1 (100.0)	2.5–100.0	0 (0.0 %)	0–10.0
	*Pseudomonas*	6 (5.3)	2.0–11.2	3 (50.0)	11.8–88.2	3 (50.0 %)	1.8–23.1
	*Escherichia*	5 (4.4)	1.5–10.0	1 (20.0)	0.5–71.6	4 (80.0 %)	3.2–26.7
	*Enterobacter*	4 (3.5)	1.0–8.8	3 (75.0)	19.4–99.4	1 (25.0 %)	0.1–14.9
Gram-negative	*Proteus*	4 (3.5)	1.0–8.8	1 (25.0)	0.6–80.6	3 (75.0 %)	1.8–23.1
	*Citrobacter*	3 (2.7)	0.6–7.6	2 (66.7)	9.4–99.2	1 (33.3 %)	0.1–14.9
	*Klebsiella*	3 (2.7)	0.6–7.6	3 (100.0)	29.2–100.0	0 (0.0 %)	0–10.0
	*Acinetobacter*	2 (1.8)	0.2–6.2	1 (50.0)	1.3–98.7	1 (50.0 %)	0.1–14.9
	*Morganella*	2 (1.8)	0.2–6.2	0 (0.0)	0.0–84.2	2 (100.0 %)	0.7–19.2
	*Cutibacterium*	2 (1.8)	0.2–6.2	1 (50.0)	1.3–98.7	1 (50.0 %)	0.1–14.9
Anaerobes	*Finegoldia*	2 (1.8)	0.2–6.2	2 (100.0)	15.8–100.0	0 (0.0 %)	0–10.0
	Clostridium	1 (0.9)	0.0–4.8	1 (100.0)	2.5–100.0	0 (0.0 %)	0–10.0
Fungi	*Candida*	8 (7.1)	3.1–13.5	3 (37.5)	8.5–75.5	5 (62.5 %)	4.8–30.3
Culture-negative		3 (2.7)	0.6–7.6	1 (33.3)	0.8–90.6	2 (66.7 %)	0.7–19.2

Abbreviations: DAA – direct anterior approach, CI: confidence interval.

## Appendix C

**Table C1:** Distribution of detected germs and obesity with 95 % confidence intervals.

	Microbial pathogen	Overall	No BMI	Obesity no – N (%)	95 % CI	Obesity yes – N (%)	95 % CI	p value	p value
				BMI < 30		BMI ≥ 30			adjusted
	*Staphylococcus*	40	0	31 (77.5)	62.5–87.7	9 (22.5)	12.3–37.5	0.962	>0.999
	*Enterococcus*	17	1	12 (75.0)	50.5–89.8	4 (25.0)	10.2–49.5	0.313	>0.999
Gram-positive	*Streptococcus*	7	1	5 (83.3)	43.6–97.0	1 (16.7)	3.0–56.4	0.101	>0.999
	*Corynebacterium*	2	1	1 (100.0)	20.7–100.0	0 (0.0)	0.0–79.3	0.026	0.338
	*Bacillus*	1	0	1 (100.0)	20.7–100.0	0 (0.0)	0.0–79.3	1	>0.999
	*Granulicatella*	1	0	1 (100.0)	20.7–100.0	0 (0.0)	0.0–79.3	1	>0.999
	*Pseudomonas*	6	0	2 (33.3)	9.7–70.0	4 (66.7)	30.0–90.3	0.077	>0.999
	*Escherichia*	5	0	5 (100.0)	56.6–100.0	0 (0.0)	0.0–43.4	0.366	>0.999
	*Enterobacter*	4	0	3 (75.0)	30.1–95.4	1 (25.0)	4.6–69.9	1	>0.999
Gram-negative	*Proteus*	4	0	0 (0.0)	0.0–49.0	4 (100.0)	51.0–100.0	0.004	0.056
	*Citrobacter*	3	0	2 (66.7)	20.8–93.9	1 (33.3)	6.1–79.2	1	>0.999
	*Klebsiella*	3	0	3 (100.0)	43.9–100.0)	0 (0.0)	0.0–56.1	0.584	>0.999
	*Acinetobacter*	2	0	1 (50.0)	9.5–90.5	1 (50.0)	9.5–90.5	0.46	>0.999
	*Morganella*	2	0	1 (50.0)	9.5–90.5	1 (50.0)	9.5–90.5	0.46	>0.999
	*Cutibacterium*	2	0	1 (50.0)	9.5–90.5	1 (50.0)	9.5–90.5	0.46	>0.999
Anaerobes	*Finegoldia*	2	0	2 (100.0)	34.2–100.0	0 (0.0)	0.0–65.8	1	>0.999
	*Clostridium*	1	0	0 (0.0)	0.0–79.3	1 (100.0)	20.7–100.0	0.263	>0.999
Fungi	*Candida*	8	0	4 (50.0)	21.5–78.5	4 (50.0)	21.5–78.5	0.098	>0.999
Culture-negative		3	0	1 (33.3)	6.1–79.2	2 (66.7)	20.8–93.9	0.189	>0.999

Abbreviations: BMI – body mass index, CI: confidence interval.
